# Reduced cytosolic carboxypeptidase 6 (CCP6) level leads to accumulation of serum polyglutamylated DNAJC7 protein: A potential biomarker for renal cell carcinoma early detection

**DOI:** 10.18632/oncotarget.8107

**Published:** 2016-03-16

**Authors:** Chong Li, Jihan Wang, Junfeng Hao, Baijun Dong, Yi Li, Xiaoxiao Zhu, Juan Ding, Shuangchun Ren, Heping Zhao, Song Wu, Yong Tian, Guo-Qing Wang

**Affiliations:** ^1^ The Key Laboratory for Bionics Engineering, Ministry of Education, College of Basic Medical Science, Jilin University, Changchun 130021, China; ^2^ Laboratory Animal Center, CAS Key Laboratory of RNA Biology, Institute of Biophysics, Chinese Academy of Sciences, Beijing 100101, China; ^3^ The Affiliated Luohu Hospital of Shenzhen University, Shenzhen Luohu Hospital Group, Shenzhen 518000, China; ^4^ Clinical Laboratory of Hong-Hui Hospital, Xi'an Jiaotong University College of Medicine, Xi'an 710054, China; ^5^ Department of Urology, Renji Hospital, School of Medicine, Shanghai Jiao Tong University, Shanghai 200127, China; ^6^ Department of Anesthesiology, Peking University Third Hospital, Beijing 100083, China

**Keywords:** renal cell carcinoma, polyglutamylation, CCP6, polyE-DNAJC7, diagnosis biomarker

## Abstract

Renal cell carcinoma (RCC) is frequently diagnosed at advanced stages of disease, although early diagnosis has much favorable prognosis. This study assessed aberrant expression of cytosolic carboxypeptidase 6 (CCP6) leading to accumulation of serum polyglutamylated DNAJC7 as a biomarker for early RCC detection. A total of 835 RCCs, 143 chronic nephritis, 170 kidney stones and 415 health controls were collected for qRT-PCR, immunohistochemistry and Western blot analysis of CCP6 expression and mass spectrometry of DNAJC7 and polyglutamylated DNAJC7. The data showed that CCP6 expression was significantly decreased in 30 RCC tissues and that mass spectrometric and pull-down analysis identified DNAJC7 as a substrate of CCP6 and showed upregulated polyglutamylated-DNAJC7 (polyE-DNAJC7) in sera of RCC patients. The electrochemiluminescence immunoassay of large-scale serum samples from multi-institutes further confirmed the remarkable increase of polyE-DNAJC7 in 805 RCCs compared to that of 385 healthy controls (*p* < 0.001), 128 patients with chronic nephritis (*p* < 0.001), and 153 with kidney stone (*p* < 0.001). Serum level of DNAJC7-polyE protein was also associated with advanced RCC stage and grade in 805 patients. The data from the current study for the first time demonstrated increased serum polyglutamylated DNAJC7 as a potential biomarker for RCC early detection and association with advanced tumor stages and grade, which provides support of further polyglutamylation research in RCC.

## INTRODUCTION

Renal cell carcinoma (RCC) originates from the lining of the proximal convoluted tubule and is the most common subtype of kidney cancer in adults. RCC accounts for approximately 90%–95% of kidney cancer cases and almost 3% of all cancer cases in the world [1, 2]. Clinically, RCC is often asymptomatic at early stage and clinical manifestations of pain, mass, and hematuria indicate an advanced or metastatic disease; thus, RCC is usually diagnosed at an advanced stage [3]. To date, early RCC diagnosis followed by nephrectomy is associated with favorable prognosis (5-year survival rate ∼85%) [4], whereas survival of patients with metastatic RCC is generally poor (5-year survival rate ∼10%) [4]. Thus, early detection of RCC, like most of other human cancers, is still the key to improve survival of RCC patients. Serum biomarker is one of biomarkers frequently investigated in our research community, which is less invasive, inexpensive, and convenient and readily applicable compared to renal biopsy or CT or ultrasound images. For example, several serum biomarkers have been evaluated for early detection of RCC, including miRNAs [5] and altered serum protein profiles [4, 6].

In this study, we evaluated protein polyglutamylation as a biomarker in early detection of RCC. Polyglutamylation is a posttranslational modification of proteins that adds glutamate side chains to proteins by tubulin tyrosine ligase-like (TTLL) enzymes [7, 8] and is deglutamylated by cytosolic carboxypeptidases (CCPs) [9]. Overexpression of TTLLs lead to accumulation of polyglutamylated-proteins, which have been recognized as critical mediator of tumorigenesis and cancer progression [10]. Altered expression of these enzymes and/or polyglutamylated-proteins contribute to cell cycle progression [11] and resistance to chemotherapy [12]. For example, a previous study showed that overexpression of TTLL4 involved in PELP1 polyglutamylation in pancreatic ductal adenocarcinoma [13]. Furthermore, DNAJ proteins belongs to the heat shock protein (HSP) 40 family and HSP40/DNAJ co-chaperones constitute the largest and most diverse sub-group of the heat shock protein (HSP) family [14, 15]. To date, 49 members in human DNAJ family were discovered and can be divided into three subclasses, DNAJA (type I), DNAJB (type II), and DNAJC (type III). The families are widely considered as regulators of HSP70 and HSP90 function [15] and the latters have been reported as key factors in cancer development and considered as targets for development of anti-cancer drugs [16, 17]. For example, DNAJB8 was shown to be a novel target of cancer stem-like cell/ cancer-initiating cell (CSC/CIC)-targeting immunotherapy of colon cancer [18]. In previous study, we demonstrated that CCP6 was required for megakaryopoiesis and *CCP6*-deficiency leading to Mad2 polyglutamylation played a crucial role in regulation of megakaryopoiesis [19]. Thus, in this study, we assessed aberrant expression of cytosolic carboxypeptidase 6 (CCP6) leading to accumulation of DNAJC7 polyglutamylation as a serum marker for early RCC detection in tissue or serum samples from a total of 835 RCCs, 143 chronic nephritis, 170 kidney stones, and 415 health controls.

## RERULTS

### Decrease in CCP6 expression in RCC tissues

**Figure 1:**
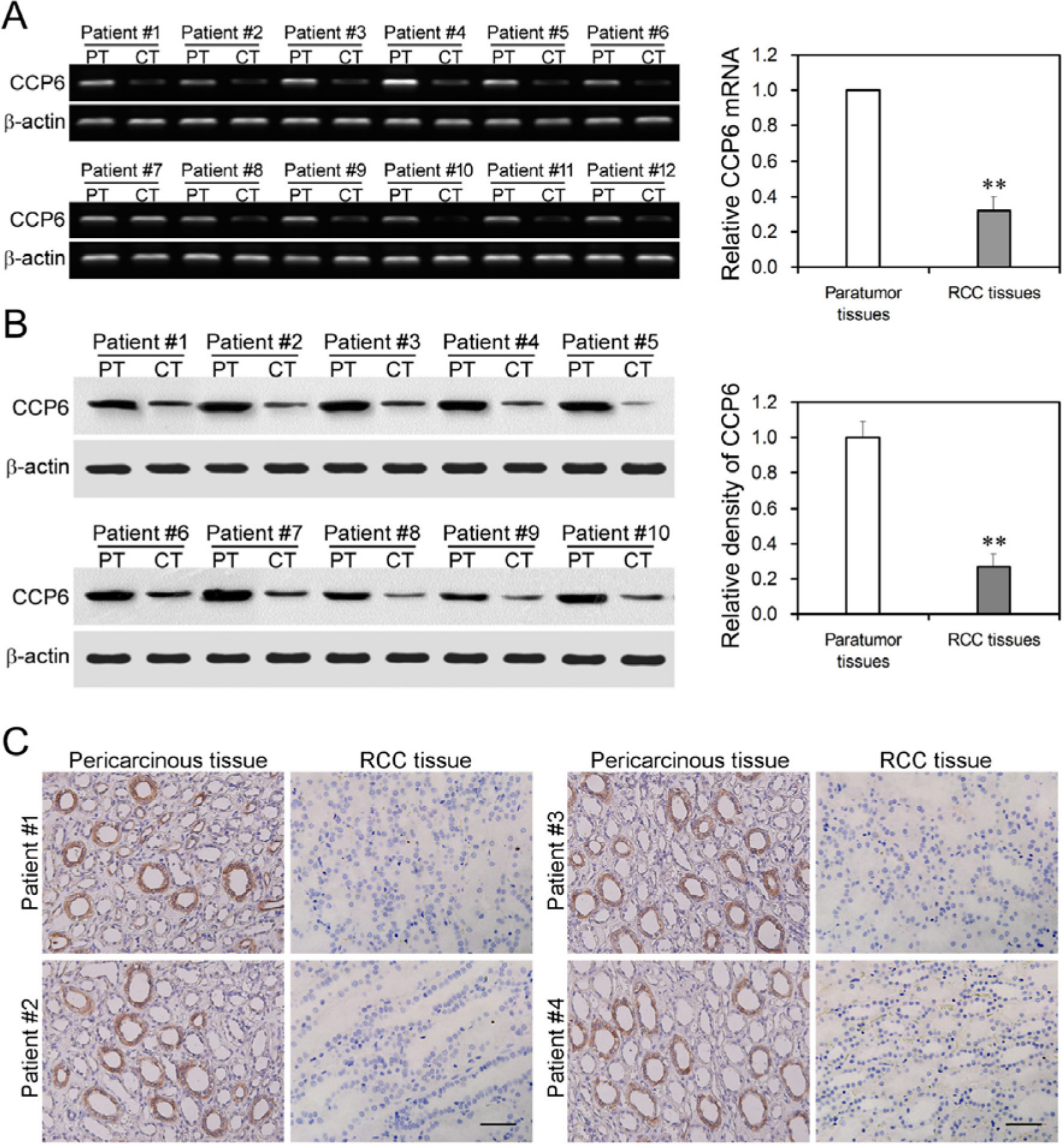
Downregulated CCP6 expression in RCC tissues (**A**) RT-PCR. Expression of CCP6 and β-actin mRNA was measured using RT-PCR in RCC cancer tissues (CT) and pericancerous tissues (PT). **P < 0.01. (**B**) Western blot analysis of CCP6 protein (58 kD) and β-actin (43 kD) expression in RCC cancer tissues (CT) and pericancerous tissues (PT). The graph is the density comparison of CCP6 protein expression (CCP6/β-actin) of RCC cancer tissues vs. pericancerous tissues. **P < 0.01. (**C**) Immunohistochemical staining of CCP6 expression in RCC cancer tissues and pericancerous tissues. (×200).

In this study, we first assessed expression level of CCP6 mRNA and protein in 30 pairs of RCC and pericancerous tissues (Table 1) and found that expression of CCP6 mRNA and protein was significantly down regulated in RCC tissues (Figure 1 and Figure S1), *P* < 0.01. CCP6 protein was expressed in the cytoplasm of pericancerous tissues but low expressed in RCC tissues (Figure 1C).

**Table 1:** Clinical characteristics of patients for ECLIA analysis

Patients' information	Test cohort				Validation cohort			
	RCC	Chronic nephritis	Kidney stone	Health control	RCC	Chronic nephritis	Kidney stone	Health control
***Age (years***)								
< 60	14	7	11	16	368	61	91	206
≥ 60	16	8	6	14	437	67	62	179
***Gender***								
Male	20	10	10	19	525	83	97	247
Female	10	5	7	11	280	45	56	138
***TNM stage***								
I	18				511			
II	6				199			
III	4				68			
IV	2				27			
***Grade stage***								
1	10				236			
2	12				351			
3	8				218			

The 30 RCC serum samples in test cohort were collected from the same patients in tissue collection part.

We further explored the expression pattern of CCP6 in different tumor samples from TCGA database (The Cancer Genome Atlas, https://genome-cancer.ucsc.edu, 2/10/2016). Results in Figure S2 indicated that mRNA of CCP6 was reduced in kidney renal cell carcinoma (KIRC) compared with control samples (*P* < 0.01), which was consistant with our results.

### DNAJC7, a novel substrate for CCP6 and polyglutamylated in RCC sera

**Figure 2:**
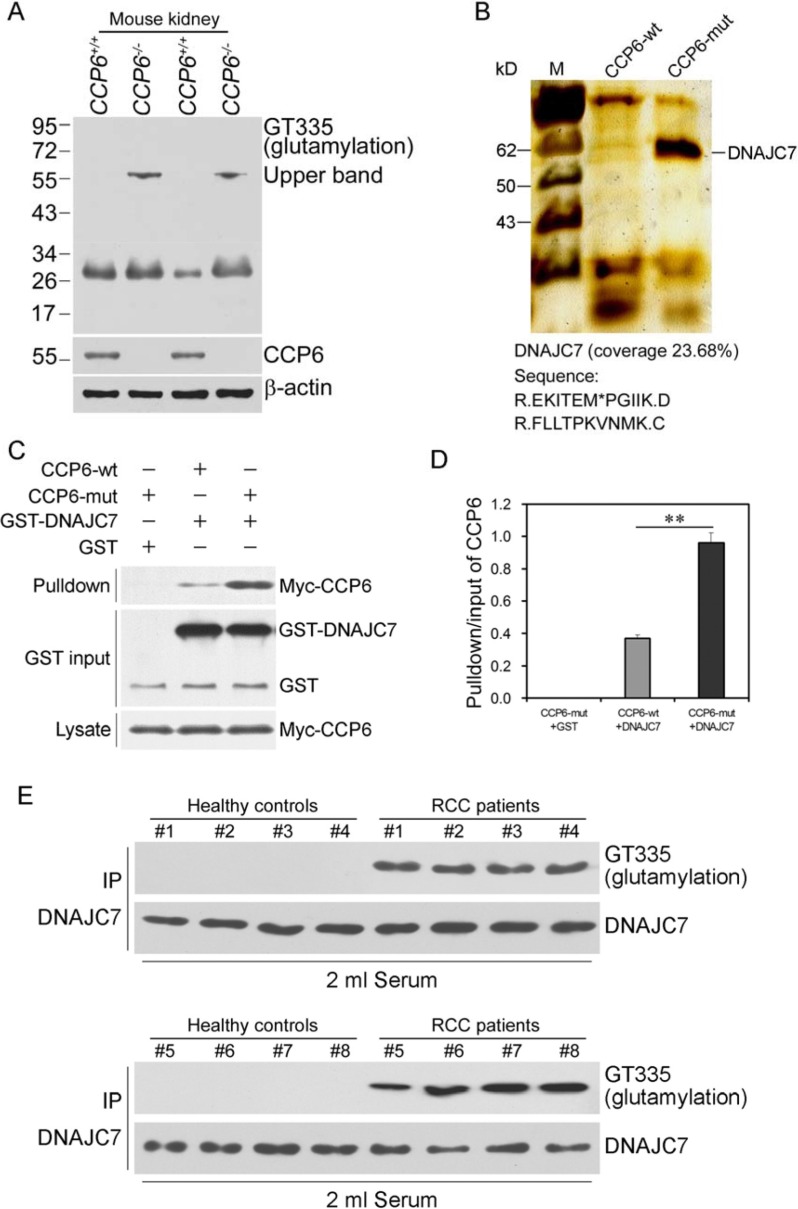
Glutamylated GST-DNAJC7 protein binding to CCP6 protein in RCC tissues and sera (**A**) Protein polyglutamylation was analyzed by immunoblotting with the GT335 antibody. Protein lysates from CCP6-deficient and the control mouse kidneys were analyzed. (**B**) CCP6-wt and CCP6-mut were immobilized with Affi-gel10 resin to go through kidney lysates for affinity chromatography. The eluted fractions were visualized by SDS-PAGE followed by silver staining. (**C**) Immunoprecipitation assay. The pull-down assays using GST-DNAJC7 incubated with the lysates from Myc-tagged CCP6-wt and Myc-tagged CCP6-mut 293T cells. Data were repeated for three times. (**D**) The pull-down/input ratios of CCP6 protein density in CCP6-wt and CCP6-mut 293 T cells. **P < 0.01. (**E**) Immunoprecipitation-Western blot. DNAJC7 protein with a glutamylated modification status in RCC sera. Immunoblotting analysis of GT335 signal in sera from RCC patients and health controls was immunoprecipitated with the anti-DNAJC7 antibody.

As a family of cytosolic carboxypeptidases, CCP6 removes the C-terminal tyrosine from substrate and deficiency of CCP6 level leads to a hyperglutamylation of the targeting protein [19]. Reduced or lost CCP6 expression could play a role in RCC development and progression; thus, we first identified CCP6 substrates in RCC tissues. CCP6-mut was immobilized to the Affi-gel10 resin and mixed with serum samples from three RCC patients, respectively. Mass spectrometry showed a consistent result that DNAJC7 was a novel candidate substrate for CCP6. (Table S1). We further analyzed lysates of kidney from CCP6-deficient mice and control mice by immunoblotting using a glutamylation-specific antibody GT335, since GT335 can recognize all glutamylation forms of a protein to detect levels of DNAJC7 polyglutamylation [19, 20]. Two blot bands around 55 kDa and 30 kDa appeared in the lanes of CCP6-deficient kidney lysates (Figure 2A), while the band around 55 kDa could not be detected in the control kidney lysates. These observations suggest the band around 55 kDa may be potential candidate substrate for CCP6. We further generated an enzymatically inactive CCP6-mut with H230S and E233Q mutations in 293T cells as reported previously [9] to identify the candidate substrate of CCP6. CCP6-wt and CCP6-mut were immobilized with Affi-gel10 resin to go through kidney lysates from both groups for affinity chromatography. The eluted fractions were visualized by SDS-PAGE followed by silver staining. Band around 55 kDa was appeared in the CCP6-mut gel and was DNAJC7 (58 kDa, Figure 2B), a novel candidate substrate for CCP6. The association of CCP6 and DNAJC7 was verified in CCP6-mut and DNAJC7 cotransfected 293T cells by a co-immunoprecipitation assay (data not shown). The glutamylated rGST-DNAJC7 could pull down Myc-tagged CCP6 protein with CCP6-mut protein possessed of a stronger bound density (Figure 2C and 2D). These data indicate that DNAJC7 is the novel substrate of CCP6 in RCC tissues.

Furthermore, we assessed level of DNAJC7 mRNA in these 30 pairs of RCC cancer vs. pericancerous tissues (Table 1) using qRT-PCR and found no significant difference in DNAJC7 mRNA level between case and control (*P* > 0.05, Figure S3). However, immunoprecipitation data showed that level of polyglutamylated-DNAJC7 protein was obviously high in RCC cancer vs. pericancerous tissues (Figure S4A) and in serum samples of RCC patients vs. healthy controls (Figure 2E and Figure S4B). The samples were first immunoprecipitated with an anti-DNAJC7 antibody followed by immunoblotting with the GT335 antibody. The results showed that sera from healthy controls showed almost no glutamylation bands, whereas the GT335 antibody detected a significant glutamylation signal in RCC sera (Figure 2E and Figure S4B), indicating that polyglutamylated form of DNAJC7 protein in RCC sera.

### Confirmation of serum polyglutamylated DNAJC7 protein as a biomarker for RCC

**Figure 3:**
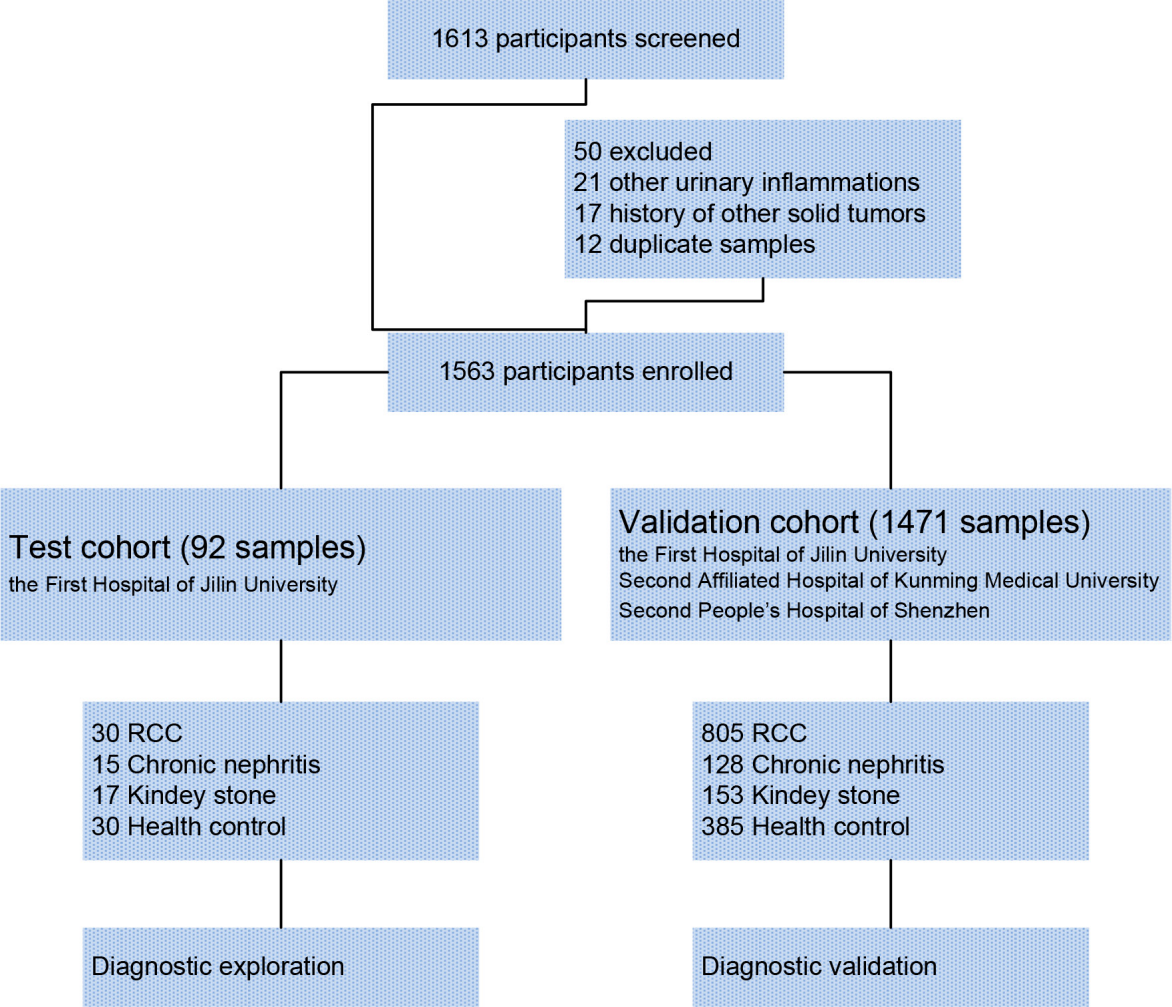
Consort diagram of the experimental design

**Figure 4:**
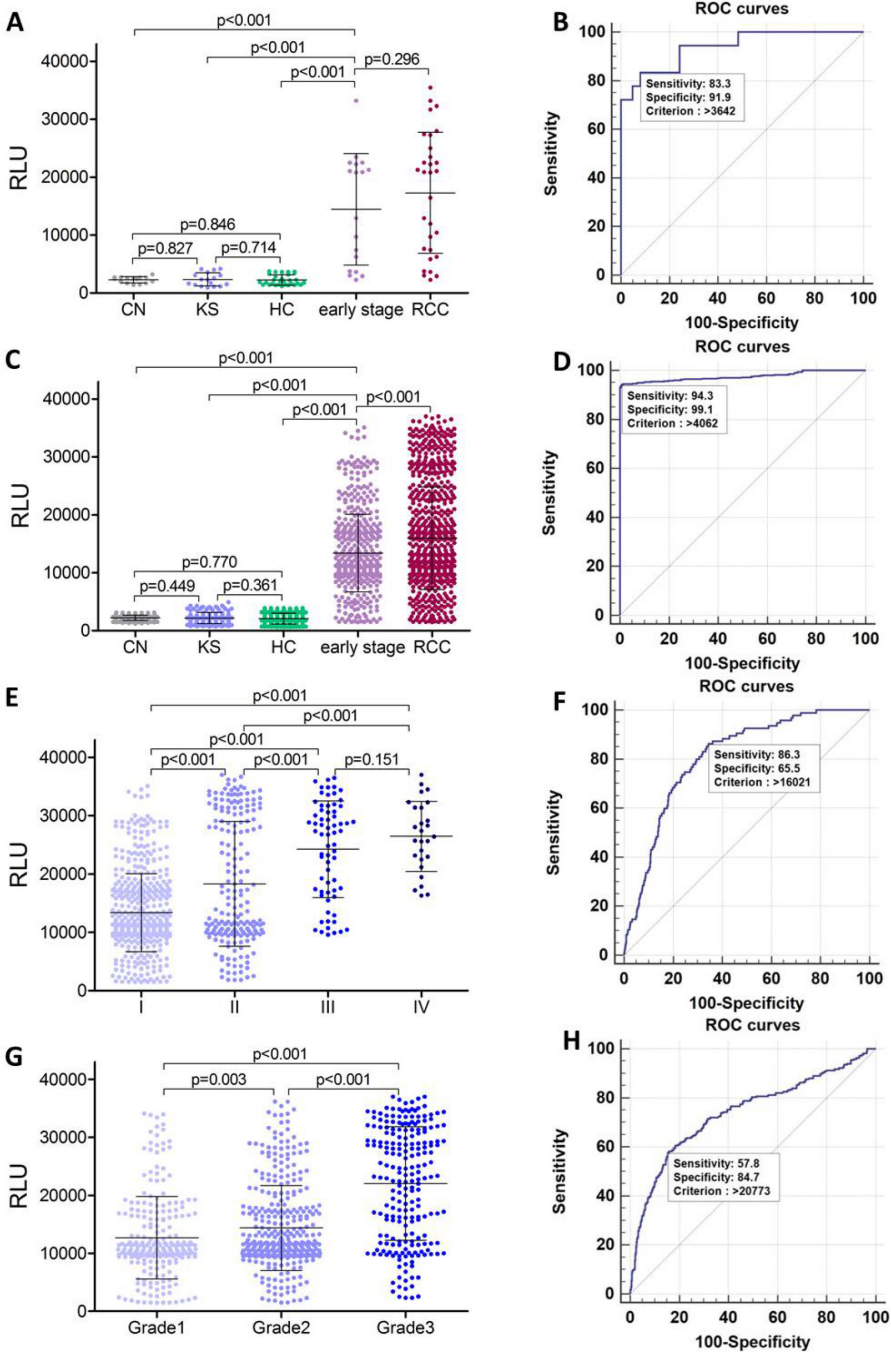
Serum concentration (RLU) and ROC analysis of polyglutamylated-DNAJC7 protein (**A**) Concentration (RLU) of serum polyglutamylated DNAJC7 protein in chronic nephritis, kidney stone patients, health controls, early stage (I) RCC and all RCC patients in the test cohort. (**B**) ROC analysis of polyglutamylated DNAJC7 protein as a discriminative biomarker between early stage (I) RCC and non-tumor samples (chronic nephritis & kidney stone patients & health controls) in the cohort. (**C**) Concentration (RLU) of serum polyglutamylated DNAJC7 protein in chronic nephritis, kidney stone patients, health controls, early stage (I) RCC and all RCC patients in validation cohort. (**D**) ROC analysis of polyglutamylated DNAJC7 protein as a discriminative biomarker between early stage (I) RCC patients and non-tumor samples (chronic nephritis & kidney stone patients & health controls) in validation cohort. (**E**) Concentration (RLU) of serum polyglutamylated DNAJC7 protein in different stages of RCC patients in validation cohort. (**F**) ROC analysis of polyglutamylated DNAJC7 protein as a discriminative biomarker between III–IV RCC and I-II RCC patients in validation cohort. (**G**) Concentration (RLU) of serum polyglutamylated DNAJC7 protein in different grades of RCC patients in validation cohort. (**H**) ROC analysis of polyglutamylated DNAJC7 protein as a discriminative biomarker between Grade3 and Grade1-2 RCC patients in validation cohort.

To confirm serum polyglutamylated DNAJC7 protein as a biomarker for RCC, we designed an electrochemiluminescence immunoassay (ECLIA) with the GT335 antibody to detect polyglutamylated DNAJC7 protein in different serum samples (Figure S5). We first assessed polyglutamylated-DNAJC7 protein using this ECLIA in sera from these 30 RCC patients, 15 chronic nephritis, 17 kidney stone patients and 30 health controls as a test cohort. After that, we obtained a large-scale cohort of serum samples from multicenter, including 805 RCC, 128 chronic nephritis, 153 kidney stone patients, and 385 health controls for further validation cohort (Figure 3 and Table 1). The average of relative light units (RLU) of polyglutamylated DNAJC7 protein was 17309 ± 10450 in 30 RCC sera of the test cohort and 15969 ± 8852 in 805 RCC sera of the validation cohort, significantly higher (*P* < 0.001) than that of chronic nephritis sera (2270 ± 541 in 15 cases of the test cohort and 2205 ± 469 in 128 cases of the validation cohort), kidney stone sera (2339 ± 1145 in 17 cases of the test cohort and 2169 ± 967 in 153 cases of the validation cohort), and health control sera (2229 ± 884 in 30 cases of the test cohort and 2086 ± 941 in 385 cases of the validation cohort; Figure 4A and 4C).

Furthermore, since more than half of RCC sera were from RCC patients with an early stage (TNM I, 18/30 in 30 cases from test cohort and 511/805 in 805 cases from validation cohort), we further observed that the RLU of serum polyglutamylated DNAJC7 protein was 14437 ± 9642 and 13396 ± 6680 in these test and validation cohorts of patients with only early stage RCC. ROC analysis indicating that the data could well separate RCC from the early stage RCC and non-tumor controls with 83.3% sensitivity and 91.9% specificity in the test cohort and 94.3% sensitivity and 99.1% specificity in the validation cohort; the cutoff criterion of RLU was 3642–4062 of these two cohorts of RCC samples (Figure 4B and 4D and Table 2). These demonstrated a potential of polyglutamylated DNAJC7 protein as a serum biomarker for early detection of RCC.

**Table 2:** Summary of the ROC analysis of polyglutamylated-DNAJC7 as discrimative biomarkers between different groups in the two sets of samples

Groups		Sensitivity(%)	Specificity(%)	AUC(95%CI)	Criterion	PPV (%)	NPV (%)	PLR	NLR	*P*-value (Area = 0.5)
Test cohort	RCC vs. CN,KS & HC	83.3	100.0	0.963 (0.902−0.991)	4200	100.0	92.5	—	0.167	< 0.0001
	early stage RCC vs. CN,KS & HC	83.3	91.9	0.939 (0.862−0.980)	3642	75.0	95.0	10.284	0.182	< 0.0001
Validationcohort	RCC vs. CN,KS & HC	94.8	99.4	0.980 (0.971−0.986)	4122	99.5	94.1	158.000	0.052	< 0.0001
	early stage RCC vs. CN,KS & HC	94.3	99.1	0.975 (0.965−0.983)	4062	98.8	95.8	104.778	0.058	< 0.0001
	III–IV vs I–II	86.3	65.5	0.809 (0.780−0.835)	16021	25.0	89.7	2.501	0.209	< 0.0001
	II vs. I	34.7	92.6	0.614 (0.577−0.650)	24914	63.9	94.0	4.689	0.705	< 0.0001
	III–IV vs. I	74.7	81.8	0.860 (0.829−0.886)	18484	43.0	94.6	4.104	0.309	< 0.0001
	III–IV vs. II	87.4	52.8	0.678 (0.621−0.631)	15164	46.6	89.7	1.852	0.239	< 0.0001
	Grade3 vs. Grade1–2	57.8	84.7	0.741 (0.709−0.771)	20773	58.1	84.4	3.778	0.498	< 0.0001
	Grade2 vs. Grade1	54.1	58.5	0.572 (0.531−0.613)	11486	66.0	46.2	1.304	0.785	0.0028
	Grade3 vs. Grade1	58.3	88.6	0.860 (0.829−0.886)	18484	76.9	69.8	5.114	0.471	< 0.0001
	Grade3 vs. Grade2	61.0	78.9	0.723 (0.684−0.760)	20773	66.3	75.8	2.891	0.494	< 0.0001

AUC: Area Under Curves; PPV: Positive Predictive Value; NPV: Negative Predictive Value; PLR: Positive Likelihood Ratio; NLR: Negative Likelihood Ratio.

### Association of polyglutamylated DNAJC7 protein with clinicopathological data from RCC patients

After that, we associated serum level of polyglutamylated-DNAJC7 protein with clinicopathological data from RCC patients in validation cohort and found that serum level of polyglutamylated DNAJC7 protein was associated with RCC TNM stages and grades (Figure 4E and 4G and Table 1). ROC curves showed a sensitivity = 86.3, specificity = 65.5, AUC = 0.809 between III–IV and I–II RCC patients (Figure 4E and Table 2) and a sensitivity = 57.8, specificity = 84.7, AUC = 0.741 between Grade 3 (poorly-differentiated) and Grade 1–2 (well and moderately-differentiated) RCCs (Figure 4H and Table 2), ROC analysis between other groups were also summarized in Table 2 and Figure S6.

## DISCUSSION

To date, there is still a great challenge in clinic to early diagnose renal cell carcinoma due to lack of effective methods to distinguish malignant tumors from benign kidney masses [21]. Although CT, MRI, or ultrasound images did have made a significant progress in recent decades in diagnosis of RCC, accuracy is still limited. Renal biopsy could improve the accuracy in certain degree, but it is an invasive procedure that always accompanied by different side effects. Recently, researchers focused on molecular biomarkers to detect RCC early or even for prediction of therapeutic effect [22, 23]. For example, detection of downregulated pro-apoptotic gene ASC/TMS1 in RCC cell lines and tissues could serve as a biomarker because it functions as a tumor suppressor in human cells. Detection of ASC/TMS1 methylation was also used as a biomarker for diagnostic and treatment responses in RCC [24]. In the current study, we assessed and identified the elevated level of serum polyglutamylated DNAJC7 protein as a biomarker for early RCC detection, especially in TNM stage I RCCs with > 80% sensitivity and specificity discriminating from non-malignant samples. Thus, our study provided us a perspective to utilize increased level of serum polyglutamylated DNAJC7 as a biomarker for RCC screening after a further validation using another cohort of serum samples.

In this study, we performed mass spectrometry and the pull-down analysis to identify the interaction between CCP6 and DNAJC7 (Table S1 and Figure S2). CCP6 was reported to be a cytosolic carboxypeptidase that catalyzes the removal of polyglutamate chains of its substrate [9] and the GT335 antibody specifically recognizes the branching point of glutamate side chains and can detect all glutamylated forms of the target proteins. Our Western blot using this glutamylation-specific antibody GT335 can detect levels of DNAJC7 polyglutamylation [19, 20], indicating that CCP6 was able to de-glutamylate DNAJC7 protein. It is true that a direct biochemical experiment could directly confirm the CCP6 enzymatic reaction to de-glutamylate DNAJC7 protein. As a type of posttranslational modification, polyglutamylation has been initially identified on α- and β-tubulin [25, 26], followed on other proteins [27–29]. However, it is just emerging in recent years for such a concept of polyglutamylation of protein in tumorigenesis and cancer progression [28]. In the current study, we utilized the ECLIA to assess serum level of polyglutamylated DNAJC7 protein in RCC sera vs. those from healthy controls, benign kidney diseases and found astonish data, i.e., while there was almost no polyglutamylated modification in sera of health controls and non-tumor kidney diseases, RCC sera showed dramatic upregulation of polyglutamylated DNAJC7 protein. Furthermore, the ECLIA is a modified ELISA and in the current study, we utilized a biotin-conjugated polyE-antibody as a detection antibody, which ensures that only DNAJC7 protein with polyglutamylated modification could be detected. This provided us a novel idea and method for polyglutamylation research in future study. Indeed, polyglutamylation of a protein causes conformational shifts of amino acids and therefore, could alter protein functions.

DNAJC7 is the member of heat shock protein 40 family, which characterized by 34 repeat domain in two n-Terminal and DNAJ domain in c-Terminal. This protein play a role of molecular chaperones by regulated heat shock protein 70 and 90 through an ATP-dependent manner [30]. A previous study demonstrated that DNAJC7 participates in the p53/MDM2-negative feedback pathway, dissociating MDM2 and p53 by inhibiting the formation of p53/MDM2 complex to improve the stability and activity of p53 protein; thus plays a role in inhibition of human carcinogenesis [31]; thus, polyglutamylated DNAJC7 would lose its function in regulation of p53 stability and activity and subsequently, RCC will occur. However, it is totally true that in the field of current cancer research, we still face a dilemma in understanding the molecular pathogenesis of RCC, like in most of human cancers. However, we do know that only in an exceptional case, alteration or mutation of one gene may not be sufficient to cause a cancer, but a gene pathway or subsequent cascades of gene alterations could play an important role in cancer development. We therefore, can't overemphasize the importance of CCP6 and DNACJ7 in RCC development. As a biomarker study, we just speculate that they may be useful in RCC early detection and further confirmation is also needed.

In summary, our current data demonstrated that elevated serum level of polyglutamylated DNAJC7 protein could be a useful biomarker in early detection of RCC, reduced expression of CCP6 could be responsible for accumulation of polyglutamylated DNAJC7 protein in RCC sera. Our current data also provided us a novel concept to understand the importance of polyglutamylation and deglutamylation in RCC development that could open an exciting field for cancer research.

## MATERIALS AND METHODS

### Clinical samples

Samples of 30 paired RCC and pericancerous tissues and sera samples, 15 serum samples of chronic nephritis, 17 serum samples of kidney stone and 30 serum samples from healthy controls (during their routine health examination) were collected from the First Hospital of Jilin University between March and October of 2013 for the test cohort. We also collected serum samples of 805 RCC, 128 chronic nephritis, and 153 kidney stone patients and 385 health controls (during their routine health examination) from the First Hospital of Jilin University, the Second Affiliated Hospital of Kunming Medical University and the Second People's Hospital of Shenzhen between March 2013 and December 2014 for further validation cohort. All renal cell carcinoma samples we choose in this study are clear cell carcinoma, and patients with RCC were confirmed by pathology and clinicopathological stage and grade were classified according to World Health Organization (WHO) criteria. Tissue samples were taken from surgery and stored in liquid nitrogen within 10 min after resection. Serum samples were centrifuged from blood and stored at −80°C until ECLIA test. This study was approved by the ethics review committee of each hospital and all participants were consented and signed a written informed consent form. All data analyses were anonymous without individual identification.

### Cell culture and vector construction

Human embryonic kidney 293T cell line was purchased from American Type Culture Collection (ATCC, Manassas, VA, USA) and cultured in Dulbecco's modified Eagle's medium (DMEM; Invitrogen, Carlsbad, CA, US) supplemented with 10% fetal bovine serum (Invitrogen) and 100 U/ml penicillin and 100 μg/ml streptomycin in a humidified incubator with 5% CO_2_ at 37°C. Human CCP6 cDNA was cloned into pCDNA4-Myc expression vector (Invitrogen) and CCP6-H230S/E233Q mutant was generated by using a site-directed mutagenesis method of DpnI digestion according to a previous study [9]. CCP6-wt and CCP6-mut cDNAs were subcloned into MBP-3C vector and purified using Amylose resin (New England BioLabs, Ipswich, MA, USA) according to the manufacturer's instructions. The Lipofectamine (Invitrogen) was utilized for gene transfection according to the manufacturer's protocol.

### qRT-PCR

Total RNA from tissue and blood samples was isolated using an RNA miniprep kit (LC Sciences, Houston, TX, USA) following the manufacturer's protocol and further reversely transcribed into cDNA with the M-MLV reverse transcriptase (Promega, Madison, WI, US). These cDNA samples were then subjected to qPCR amplification using the ABI 7300 qPCR system with primer sequences of β-actin (5′-GTCACCAACTGGGACGACAT-3′ and 5′-AGGGATAGCACAGCCTGGAT-3′, 200 bp), CCP6 (5′-CGCTTCCGAGTCTGGTTCAA-3′ and 5′-CCATAGGGGCCATCCCATCT-3′, 157 bp), or DNAJC7 (5′-GGTAATCGAGCAGCCACCTT-3′ and 5′-CTCTCTGGAAGCTGCGACAT-3′,169 bp) according to a previous study [18]. The quantitation of mRNA level was to normalize to β-actin mRNA level using a comparative Ct method (2^−ΔΔCt^, ΔCt = Ct _target_^−^ Ct_β-actin_, ΔΔCt = ΔCt_tumor_^−^ ΔCt_normal_).

### Western blotting

Approximately 1 mm^3^ of each tissue samples were retrieved from liquid nitrogen and grinded and homogenized in a cell lysis buffer (Beyotime, Beijing, China) and then centrifuged at 10000 rpm for 30 min 4°C to remove cell debris. A Bradford protein assay kit (Bio-Rad, Hercules, CA, US) was used to quantify the protein concentration and sodium dodecyl sulfate-polyacrylamide gel electrophoresis (SDS-PAGE) was to separated proteins in 12% SDS-PAGE gel and transferred onto PVDF membranes (Beyotime, Beijing, China). After 2 h 5% milk blocking, the PVDF membranes were incubated with an anti-CCP6 (Santa Cruz Biotechnology, Santa Cruz, CA, US) or anti-β-actin antibody (Sigma-Aldrich, St Louis, MO, USA) at 4°C overnight. On the next day, the membranes were further incubated with a goat anti-Rat IgG (Santa Cruz Biotechnology) for 2 h at the room temperature and then incubated with a chemiluminescence (ECL; Beyotime) kit to detect protein signals. The CCP6 protein density in each sample was normalized to the level of β-actin. Data and figures were analyzed with SPSS 18.0 software and GraphPad Prism Version 5.0, differences between groups were statistically evaluated by sample one-tailed Student's *t*-test, *p* value < 0.05.

### Immunohistochemistry

RCC and pericancerous tissues were fixed in 4% paraformaldehyde for 12^−^16 h and embedded into paraffin and cut into 4 μm sections. For immunohistochemistry, the sections were baked at 60°C for 30 min and deparaffinized in xylene for 30 min, rehydrated in series of ethanol solutions. The sections were then subjected to antigen retrieval in the citric acid buffer in microwave and to H_2_O_2_ treatment for 30 min at the room temperature. After that, the sections were incubated in phosphatebuffered saline (PBS) containing 5% porcine serum and then with a CCP6 antibody (Santa Cruz Biotechnology) at 4°C for overnight. On the next day, the sections were washed three times with PBS and further incubated with a secondary antibody for 30 min at the room temperature and then with a color reagent diaminobenzidine (Bios, Beijing, China) for visualizing of positive signal. All sections were counterstained with Mayer's hematoxylin and evaluated and scored by two pathologists (without knowledge of any of patients' information), independently under a microscope. Cells of all sections were observed at ×200 magnification and positively staining cells were identified.

### Mass spectrometry identification of DNAJC7 protein

CCP6-mut was immobilized with Affi-gel10 resin and then mixed with mouse basal membrane (BM) lysates for affinity chromatography as described previously [32]. The eluted fractions of affinity chromatography samples were visualized by SDS-PAGE followed by silver staining. Differential bands from SDS-PAGE gels were digested with trypsin and then subjected to mass spectrometry analyses with nano LC-ESILTQ MS/MS (Thermo Scientific, Waltham, MA, USA).

### Immunoprecipitation assay

Serum samples from RCC patients and controls were incubated with the anti-DNAJC7 antibody (Santa Cruz Biotechnology) and then immunoprecipitated with protein A/G agarose beads and followed by immunoblotting with the GT335 (anti-glutamylation) antibody [20] (AdipoGen, San Diego, CA, USA).

### Preparation of ECLIA plates and test

96-well Nunc-Immuno microtitre plates with MaxiSorp surface (Nalge Nunc, Penfield, NY, USA) were coated with 100 μl of 100 ng/ml solution of Anti-DNAJC7 antibody (Santa Cruz Biotechnology) in 50 mM carbonate buffer pH 9.5 and then incubated at the room temperature overnight. The plates were then washed and blocked with PBS containing 1% bovine serum albumin and 1% gelatin for 2 h at 37°C. For assessing serum samples using these plates, the plates were washed three times with PBS containing 0.05% Tween-20 (PBST). Subsequently, 50 μl of serum samples, biotin-GT335 (10 ng/ml) and streptavidin-HRP (1 ng/ml) were added to each well and incubated for 1 h at 37°C. After washing with PBST three times, the plates were dried at the room temperate. 100 μl of freshly prepared substrate solution was added to each well and stirred. Chemiluminescence intensity was then measured using a chemiluminescence apparatus (Hamamatsu, Japan).

### Statistical analysis

ECLIA data were summarized as mean ± SE and statistically analyzed by using GraphPad Prism Version 5.0 and SPSS Statistics 21 software. Difference between tumor and normal tissues were evaluated by the two-tailed *t*-test and Mann-Whitney *U* test with *p* value < 0.05 was considered to be statistically significant. Med-Calc statistical software was used to analyze Receiver Operating Characteristic (ROC) curve and the sensitivity, specificity, area under the curve (AUC) with 95% CI and criterion were automatically calculated from the Med-Calc, *p* value < 0.01 was considered to be statistically significant.

## SUPPLEMENTARY MATERIALS FIGURES AND TABLE



## References

[1] Hongo F, Takaha N, Oishi M, Ueda T, Nakamura T, Naitoh Y, Naya Y, Kamoi K, Okihara K, Matsushima T, Nakayama S, Ishihara H, Sakai T (2014). CDK1 and CDK2 activity is a strong predictor of renal cell carcinoma recurrence. Urologic oncology.

[2] Siegel R, Ma J, Zou Z, Jemal A (2014). Cancer statistics, 2014. CA.

[3] Cohen HT, McGovern FJ (2005). Renal-cell carcinoma. The New England journal of medicine.

[4] Zhao Z, Wu F, Ding S, Sun L, Liu Z, Ding K, Lu J (2015). Label-free quantitative proteomic analysis reveals potential biomarkers and pathways in renal cell carcinoma. Tumour biology.

[5] Zhao A, Li G, Peoc'h M, Genin C, Gigante M (2013). Serum miR-210 as a novel biomarker for molecular diagnosis of clear cell renal cell carcinoma. Experimental and molecular pathology.

[6] White NM, Masui O, Desouza LV, Krakovska O, Metias S, Romaschin AD, Honey RJ, Stewart R, Pace K, Lee J, Jewett MA, Bjarnason GA, Siu KW (2014). Quantitative proteomic analysis reveals potential diagnostic markers and pathways involved in pathogenesis of renal cell carcinoma. Oncotarget.

[7] Janke C, Rogowski K, Wloga D, Regnard C, Kajava AV, Strub JM, Temurak N, van Dijk J, Boucher D, van Dorsselaer A, Suryavanshi S, Gaertig J, Edde B (2005). Tubulin polyglutamylase enzymes are members of the TTL domain protein family. Science (New York, NY).

[8] van Dijk J, Rogowski K, Miro J, Lacroix B, Edde B, Janke C (2007). A targeted multienzyme mechanism for selective microtubule polyglutamylation. Molecular cell.

[9] Rogowski K, van Dijk J, Magiera MM, Bosc C, Deloulme JC, Bosson A, Peris L, Gold ND, Lacroix B, Bosch Grau M, Bec N, Larroque C, Desagher S (2010). A family of protein-deglutamylating enzymes associated with neurodegeneration. Cell.

[10] Das V, Kanakkanthara A, Chan A, Miller JH (2014). Potential role of tubulin tyrosine ligase-like enzymes in tumorigenesis and cancer cell resistance. Cancer letters.

[11] Brants J, Semenchenko K, Wasylyk C, Robert A, Carles A, Zambrano A, Pradeau-Aubreton K, Birck C, Schalken JA, Poch O, de Mey J, Wasylyk B (2012). Tubulin tyrosine ligase like 12, a TTLL family member with SET- and TTL-like domains and roles in histone and tubulin modifications and mitosis. PloS one.

[12] Kavallaris M (2010). Microtubules and resistance to tubulin-binding agents. Nature reviews Cancer.

[13] Kashiwaya K, Nakagawa H, Hosokawa M, Mochizuki Y, Ueda K, Piao L, Chung S, Hamamoto R, Eguchi H, Ohigashi H, Ishikawa O, Janke C, Shinomura Y (2010). Involvement of the tubulin tyrosine ligase-like family member 4 polyglutamylase in PELP1 polyglutamylation and chromatin remodeling in pancreatic cancer cells. Cancer research.

[14] Sterrenberg JN, Blatch GL, Edkins AL (2011). Human DNAJ in cancer and stem cells. Cancer letters.

[15] Stark JL, Mehla K, Chaika N, Acton TB, Xiao R, Singh PK, Montelione GT, Powers R (2014). Structure and function of human DnaJ homologue subfamily a member 1 (DNAJA1) and its relationship to pancreatic cancer. Biochemistry.

[16] Shevtsov MA, Komarova EY, Meshalkina DA, Bychkova NV, Aksenov ND, Abkin SV, Margulis BA, Guzhova IV (2014). Exogenously delivered heat shock protein 70 displaces its endogenous analogue and sensitizes cancer cells to lymphocytes-mediated cytotoxicity. Oncotarget.

[17] Schulz R, Moll UM (2014). Targeting the heat shock protein 90: a rational way to inhibit macrophage migration inhibitory factor function in cancer. Current opinion in oncology.

[18] Morita R, Nishizawa S, Torigoe T, Takahashi A, Tamura Y, Tsukahara T, Kanaseki T, Sokolovskaya A, Kochin V, Kondo T, Hashino S, Asaka M, Hara I (2014). Heat shock protein DNAJB8 is a novel target for immunotherapy of colon cancer-initiating cells. Cancer science.

[19] Ye B, Li C, Yang Z, Wang Y, Hao J, Wang L, Li Y, Du Y, Hao L, Liu B, Wang S, Xia P, Huang G (2014). Cytosolic carboxypeptidase CCP6 is required for megakaryopoiesis by modulating Mad2 polyglutamylation. The Journal of experimental medicine.

[20] Wolff A, de Nechaud B, Chillet D, Mazarguil H, Desbruyeres E, Audebert S, Edde B, Gros F, Denoulet P (1992). Distribution of glutamylated alpha and beta-tubulin in mouse tissues using a specific monoclonal antibody, GT335. European journal of cell biology.

[21] Verma SK, Mitchell DG, Yang R, Roth CG, O'Kane P, Verma M, Parker L (2010). Exophytic renal masses: angular interface with renal parenchyma for distinguishing benign from malignant lesions at MR imaging. Radiology.

[22] Logothetis CJ, Gallick GE, Maity SN, Kim J, Aparicio A, Efstathiou E, Lin SH (2013). Molecular classification of prostate cancer progression: foundation for marker-driven treatment of prostate cancer. Cancer discovery.

[23] Modur V, Hailman E, Barrett JC (2013). Evidence-based laboratory medicine in oncology drug development: from biomarkers to diagnostics. Clinical chemistry.

[24] Liu Q, Jin J, Ying J, Cui Y, Sun M, Zhang L, Fan Y, Xu B, Zhang Q (2015). Epigenetic inactivation of the candidate tumor suppressor gene ASC/TMS1 in human renal cell carcinoma and its role as a potential therapeutic target. Oncotarget.

[25] Edde B, Rossier J, Le Caer JP, Desbruyeres E, Gros F, Denoulet P (1990). Posttranslational glutamylation of alpha-tubulin. Science (New York, NY).

[26] Rudiger M, Plessman U, Kloppel KD, Wehland J, Weber K (1992). Class II tubulin, the major brain beta tubulin isotype is polyglutamylated on glutamic acid residue 435. FEBS letters.

[27] Wu HY, Rong Y, Correia K, Min J, Morgan JI (2015). Comparison of the enzymatic and functional properties of three cytosolic carboxypeptidase family members. The Journal of biological chemistry.

[28] Wasylyk C, Zambrano A, Zhao C, Brants J, Abecassis J, Schalken JA, Rogatsch H, Schaefer G, Pycha A, Klocker H, Wasylyk B (2010). Tubulin tyrosine ligase like 12 links to prostate cancer through tubulin posttranslational modification and chromosome ploidy. International journal of cancer.

[29] Regnard C, Desbruyeres E, Huet JC, Beauvallet C, Pernollet JC, Edde B (2000). Polyglutamylation of nucleosome assembly proteins. The Journal of biological chemistry.

[30] Moffatt NS, Bruinsma E, Uhl C, Obermann WM, Toft D (2008). Role of the cochaperone Tpr2 in Hsp90 chaperoning. Biochemistry.

[31] Kubo N, Wu D, Yoshihara Y, Sang M, Nakagawara A, Ozaki T (2013). Co-chaperon DnaJC7/TPR2 enhances p53 stability and activity through blocking the complex formation between p53 and MDM2. Biochemical and biophysical research communications.

[32] Fan Z, Beresford PJ, Oh DY, Zhang D, Lieberman J (2003). Tumor suppressor NM23-H1 is a granzyme A-activated DNase during CTL-mediated apoptosis, and the nucleosome assembly protein SET is its inhibitor. Cell.

