# Supplementary material for: Reduced cytosolic carboxypeptidase 6 (CCP6) level leads to accumulation of serum polyglutamylated DNAJC7 protein: A potential biomarker for renal cell carcinoma early detection
Source: Oncotarget. 2016 Mar 16;7(16):22385–96. doi: 10.18632/oncotarget.8107 (PMC5008367; doi:10.18632/oncotarget.8107)
Supplement: Supplementary file 1 [file oncotarget-07-22385-s001.pdf]

Reduced cytosolic carboxypeptidase 6 (CCP6) level leads to accumulation of serum polyglutamylated DNAJC7 protein: A potential biomarker for renal cell carcinoma early detection

Supplementary Materials

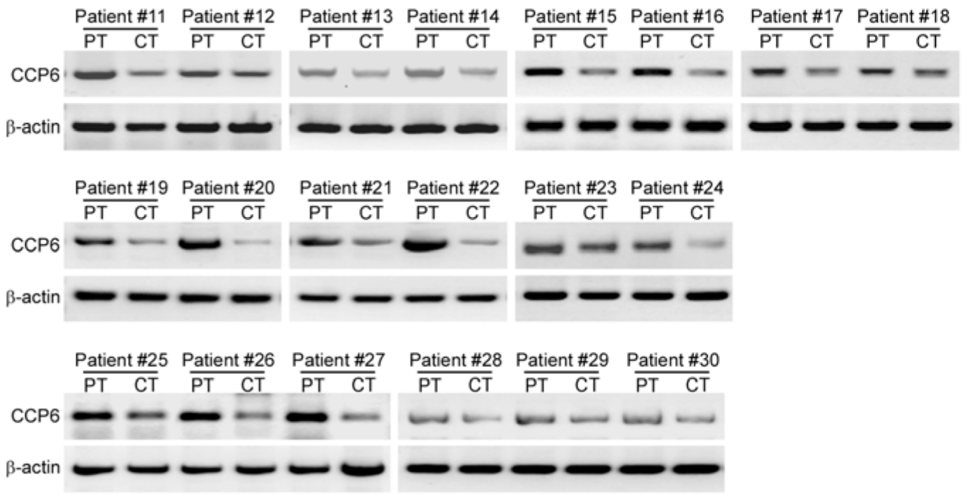

Supplementary Figure S1: Western blot analysis of CCP6 protein and β-actin expression in RCC cancer tissues (CT) and pericancerous tissues (PT).

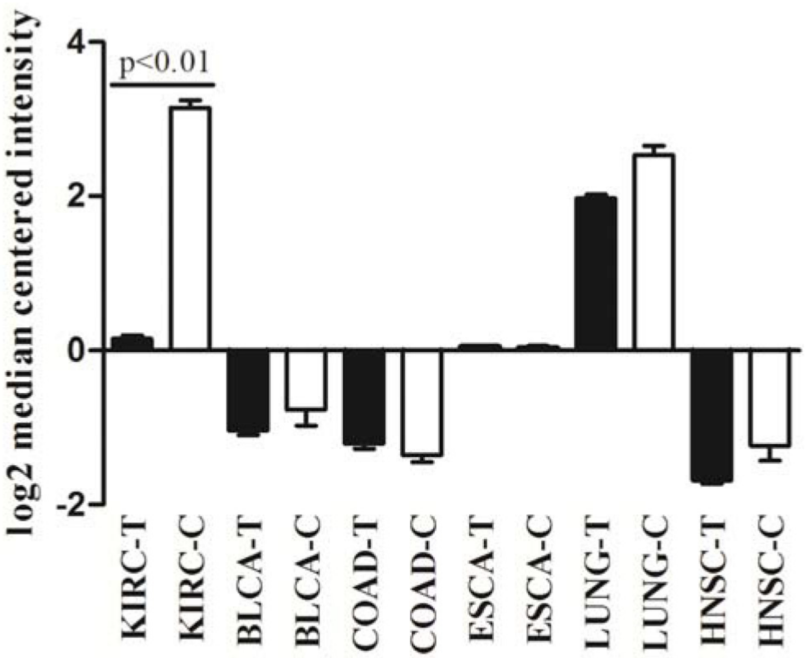

Supplementary Figure S2: CCP6 expression patten in different kind of malignancy based on TCGA database. 27 datasets of different tumor samples were downloaded from TCGA database (The Cancer Genome Atlas, https://genome-cancer.ucsc.edu) in February 2016, here summarized 6 of the 27 datasets, indicated a reduced mRNA of CCP6 in renal cell carcinoma ($P < 0.01$). KIRC: kidney clear cell carcinoma, BLCA: bladder urothelial carcinoma, COAD: colon adenocarcinoma, ESCA: esophageal carcinoma, LUNG: lung cancer, HNSC: head & neck squamous cell carcinoma.

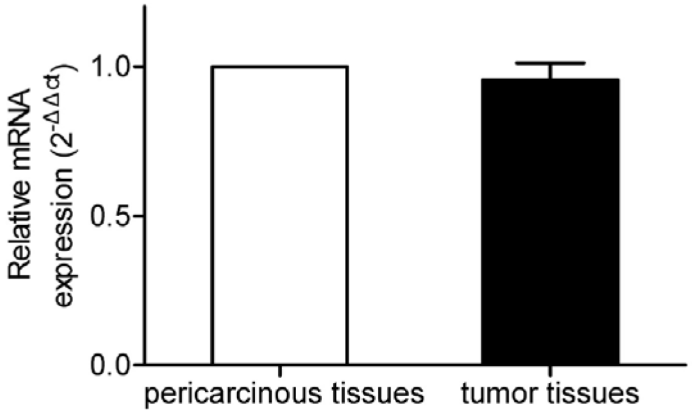

Supplementary Figure S3: DNAJC7 mRNA shows no significant difference between RCC cancer vs. pericancerous tissues. 30 pairs of RCC tumor vs. pericancerous tissues were performed for CCP6 mRNA examination. Result shows the relative fold change ($2^{-\Delta\Delta Ct}$) of DNAJC7 mRNA in RCC cancer tissues vs. pericancerous tissues.

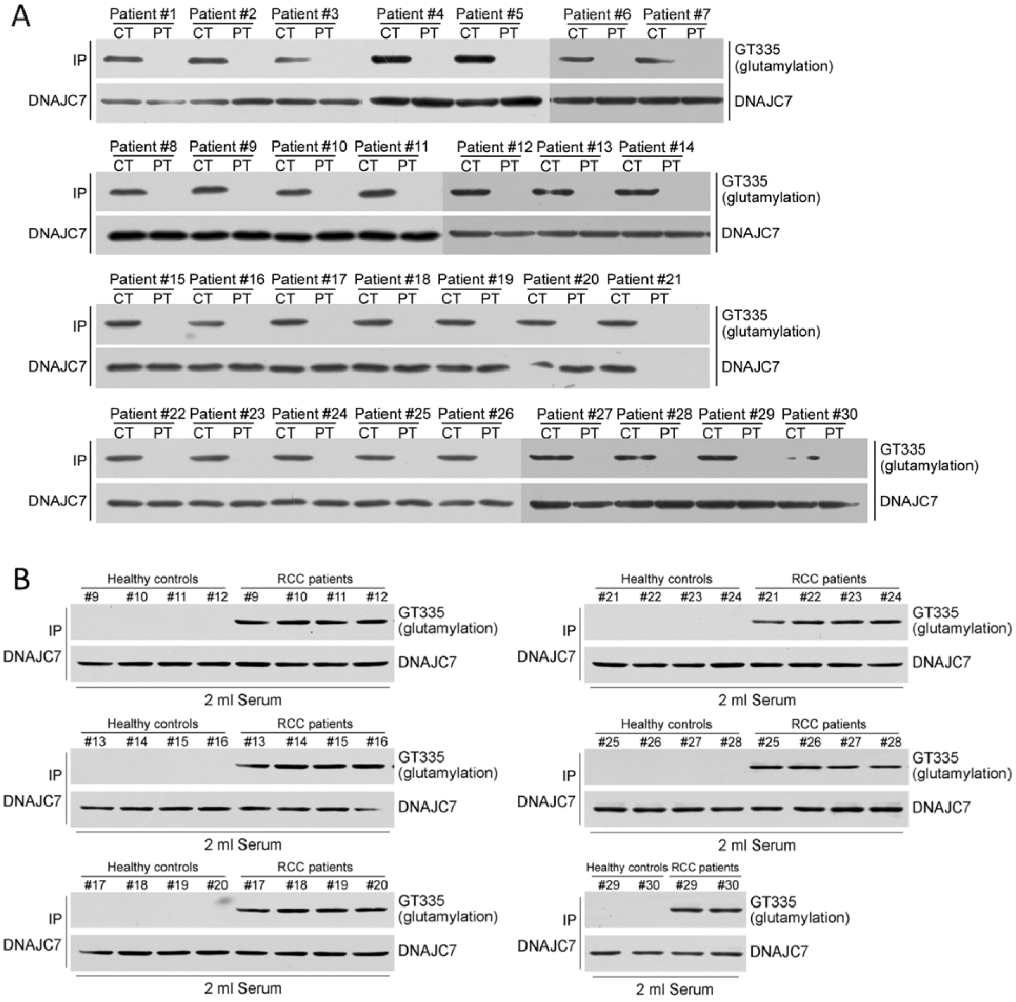

Supplementary Figure S4: Immunoprecipitation-Western blot detection of polyglutamylated-DNAJC7 levels in RCC cancer vs. pericancerous tissues and in cancer vs. healthy control sera. (**A**) RCC cancer tissues (CT) and pericancerous tissues (PT). (**B**) serum samples from RCC patients and healthy controls.

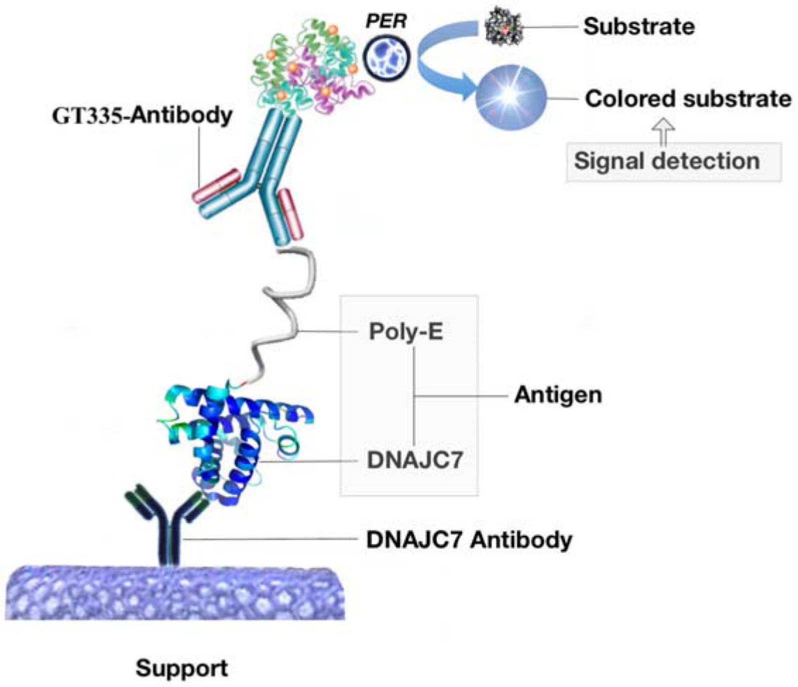

Supplementary Figure S5: Mechanism model of the electrochemiluminescence immunoassay (ECLIA) for detecting the polyglutamylated-DNAJC7 protein in serum samples. In the ECLIA analysis, DNAJC7 antibody was used as captured antibody and biotin-conjugated GT335-antibody as detection antibody.

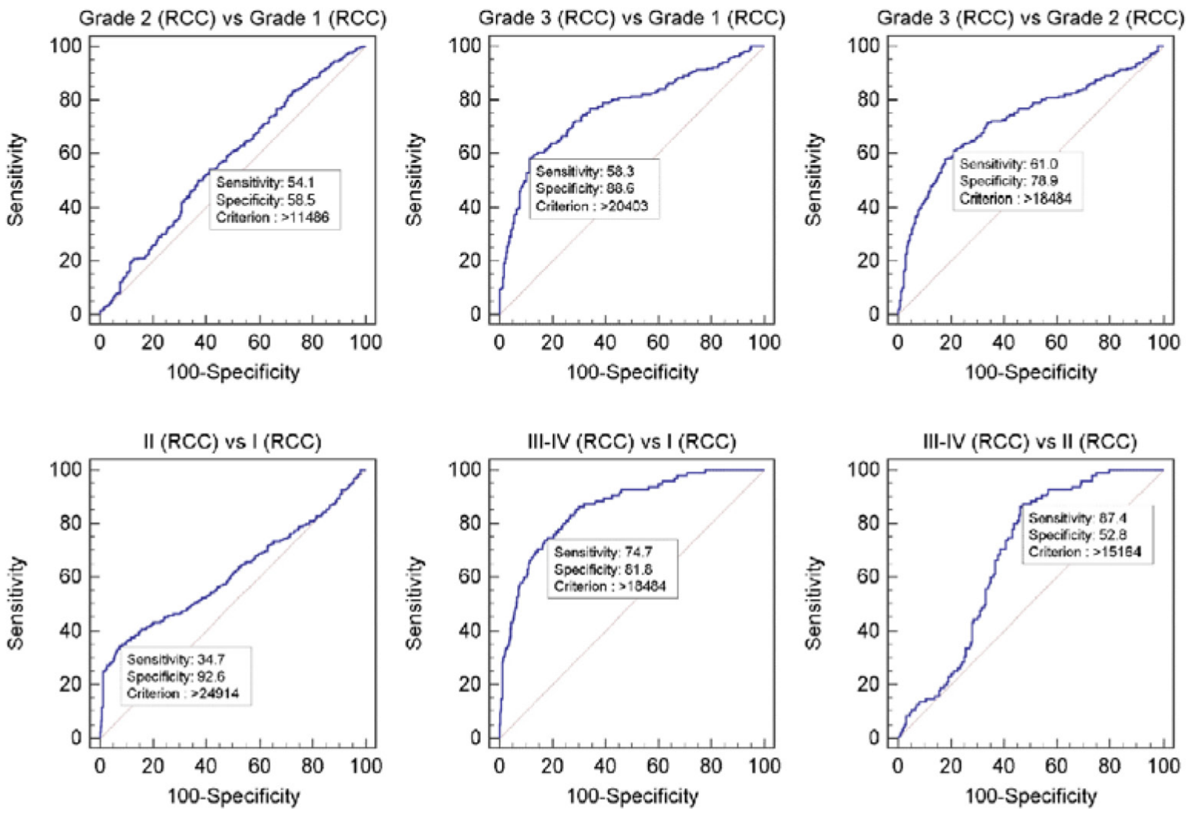

Supplementary Figure S6: ROC analysis of polyglutamylated-DNAJC7 as a discriminative biomarker between different groups in the validation cohort.

Supplementary Table S1: Complete information of the detected peptides by mass spectrometry

Reference		Score	Coverage	MW	Accession	Peptide (Hits)
**Scan(s)**	**Peptide**	**XC**	**DeltaCn**	**Sp**	**RSp**	**Ions**
DnaJ(Hsp40)homolog, subfamily C, member 7		18.14		254437.6	247494234	2 (11000)
3327	R.EKITEM * PGIIK.D	2.75	0.08	1337.2	1	16/20
8689	R.FLLTPKVNMK.C	1.89	0.06	648.1	1	11/18
DnaJ(Hsp40)homolog, subfamily C, member 7		18.14		254437.6	247494234	2 (11000)
3327	R.EKITEM * PGIIK.D	2.75	0.08	1337.2	1	16/20
8689	R.FLLTPKVNMK.C	1.89	0.06	648.1	1	11/18
DnaJ(Hsp40)homolog, subfamily C, member 7		18.14		254437.6	247494234	2 (11000)
3327	R.EKITEM * PGIIK.D	2.75	0.08	1337.2	1	16/20
8689	R.FLLTPKVNMK.C	1.89	0.06	648.1	1	11/18
eosinophil peroxidase precursor		20.15		81379.6	145966840	2 (20000)
4470	R.IPCFLAGDTR.S	2.91	0.48	1147.9	1	15/18
7062	R.VANVFTLAFR.F	2.42	0.36	1055.3	1	15/18
uncharacterized protein LOC239673		18.16		58223.9	269914154	2 (11000)
6453	K.LALDIEIATYR.R	3.27	0.59	1244.0	1	16/20
7517	R.SLNLDSIIAEVK.A	3.07	0.58	1619.7	1	18/22
